# RESTful Discovery and Eventing for Service Provisioning in Assisted Living Environments

**DOI:** 10.3390/s140509227

**Published:** 2014-05-23

**Authors:** Jorge Parra, M. Anwar Hossain, Aitor Uribarren, Eduardo Jacob

**Affiliations:** 1 Embedded Systems, IK4-Ikerlan, J.M. Arizmendiarrieta 2, Arrasate-Mondragón 20500, Spain; E-Mail: auribarren@ikerlan.es; 2 College of Computer and Information Sciences, King Saud University, PO Box 51178, Riyadh 11543, Saudi Arabia; E-Mail: mahossain@ksu.edu.sa; 3 Communications Engineering Department, University of the Basque Country (UPV/EHU), Alda. Urquijo s/n, Bilbao 48013, Spain; E-Mail: Eduardo.Jacob@ehu.es

**Keywords:** REST, middleware, discovery, eventing, health monitoring

## Abstract

Service provisioning in assisted living environments faces distinct challenges due to the heterogeneity of networks, access technology, and sensing/actuation devices in such an environment. Existing solutions, such as SOAP-based web services, can interconnect heterogeneous devices and services, and can be published, discovered and invoked dynamically. However, it is considered heavier than what is required in the smart environment-like context and hence suffers from performance degradation. Alternatively, REpresentational State Transfer (REST) has gained much attention from the community and is considered as a lighter and cleaner technology compared to the SOAP-based web services. Since it is simple to publish and use a RESTful web service, more and more service providers are moving toward REST-based solutions, which promote a resource-centric conceptualization as opposed to a service-centric conceptualization. Despite such benefits of REST, the dynamic discovery and eventing of RESTful services are yet considered a major hurdle to utilization of the full potential of REST-based approaches. In this paper, we address this issue, by providing a RESTful discovery and eventing specification and demonstrate it in an assisted living healthcare scenario. We envisage that through this approach, the service provisioning in ambient assisted living or other smart environment settings will be more efficient, timely, and less resource-intensive.

## 1. Introduction

Ambient Assisted Living (AAL) environments are electronically augmented surroundings with sensor and actuator devices, and are aware of the presence of people to provide them timely and relevant services for better health and wellness support [1]. With the increase of the elderly population, more and more of them are being placed in these AAL environments to receive caregiver assistance in activities of daily living (ADL) and health monitoring. Nowadays, it is common in AAL facilities that there are several health care devices and sensors with varying level of computational and networking capabilities. As a result, it is possible to interconnect these devices in order to realize some distributed and collaborative applications for better healthcare support in AAL environments, however, service provisioning in such environments faces distinct challenges due to the heterogeneity of networks, access technology, and sensing/actuation devices.

Several approaches and technologies have been made available in order to address the above challenges, such as OSGi [2], Web Services [3], UPnP [4] and others. Among these, web services have gained much popularity for developing interoperable systems and applications. However, this solution is considered heavier than what is required in the smart environment-like context and hence suffers from performance degradation.

As an alternative, REST, which is considered as a lighter and cleaner technology compared to the SOAP-based web services, has gained much attention from the community. Besides, it is simple to publish and use a RESTful web service. As a result, more and more service providers are moving toward REST-based solutions, which promote a resource-centric model compared to a service-centric model. However, an adequate dynamic service discovery for RESTful web services is still a challenge [5]. For AAL, it is important to work on this challenge because the addition or removal of a new sensor or service needs to be propagated dynamically to all the other entities so that they can adjust their behavior. Another challenge comes with this discovery mechanism, which is the eventing (event management) because it is also an essential feature in a sensory environment. Event management enables engagement and notifications among existing services for supporting functional collaboration and composition. This paper essentially addresses the above two challenges.

This work focuses on specifying a lightweight middleware approach based on REST for enabling dynamic discovery and event management of networked resources. More specifically, it defines a mechanism to dynamically discover sensor and actuator devices and exchange notifications among them following the REST principles. Such a middleware is suitable for service provisioning in AAL because it simplifies the development of health and wellness applications on top it.

The remainder of this paper is organized as follows: in Section 2 we comment on some related literature. Section 3 elaborates on the proposed middleware highlighting the details of the discovery and eventing mechanism. Then, Section 4 evaluates the proposed solution and provides implementation details. Finally, Section 5 concludes the paper with a note on some future work directions.

## 2. Related Work

This section briefly comments on some related literature that studies the dynamic service provisioning issue using different mechanisms, such as OSGi, DPWS and UPnP. Some of the earlier works that motivate the use of REST to interconnect devices are [6–8]. The authors in [6] describe the data and services as resources like what REST promotes. The work in [7] focuses on the discovery mechanism, which is described to be based on a resource repository (centralized). Sensors and actuators advertise themselves to this repository using the ATOM protocol. The repository also accepts queries from potential clients of the resources. The authors in [8] propose a systematic implementation of the RESTful constraints in order to expose the real-world data and functionality on the web through REST interface. The authors also state the challenge underlying the dynamic discovery for RESTful resources.

The work in [2] is a demonstration of a home care environment, which is based on the OSGi framework. This provides a platform-centric approach such that the devices and sensors are all connected to an OSGi gateway, where the application layer acts as a centralized controller to invoke and/or compose different services for the user. The dynamic discovery in OSGi is supported by local lookup service, whereas the current paper proposes RESTful discovery in a distributed dynamic environment. Although the approach in [2] provides a local solution to expose the devices as services, it requires external mechanisms such as Web Service, UPnP or REST to intercommunicate among the different OSGi platforms.

In [9], the authors propose a context sensitive web service search mechanism, adding semantics to the WSDL descriptions of the services. However they do not address the dynamic capabilities offered by other WS family of technologies such as WS-Discovery and WS-Eventing, which are comparable technologies to the proposed solutions given in our paper.

In [10,11], and in our earlier work [3], the authors justify the use of Device Profile for Web Services (DPWS) for service oriented communications to have dynamic Web Service infrastructures. Here, the devices can be discovered, described, and subscribed using Web Service-based standard protocols, such as WS-Discovery and WS-Eventing. However this set of protocols is heavier than a RESTful approach, which we analyze and show in this paper.

Compared to SOAP/XML Web Service solutions, RESTful architecture has shown its strength [12], which can be applicable to the device networking domain. REST leverages existing well-known standards (HTTP, XML, URI, MIME) and the necessary infrastructure has already become pervasive and is currently available in many networked devices. HTTP clients and servers are available for all major hardware platforms, and thus, such a lightweight infrastructure can be incorporated to a number of networked devices. Using URIs and hyperlinks, it is possible to discover resources without a centralized registry or repository approach, and hence, performance can be optimized in devices with low processing capabilities using lightweight formats for resource representations. The simplicity and easy-to-understand design guidelines make RESTful architecture suitable to be applied in consumer device domain. Furthermore, RESTful architectures are more scalable than the well-known Service Oriented Architectures (SOA) due to the uniform interface (e.g., GET, POST, PUT, DELETE). Interaction with SOA services requires understanding both data and service interface contract, while in REST only the data contract must be understood because the interface is uniform for all the services. Furthermore, HTTP support for data types in Accept and Content-Type HTTP headers also helps with scalability [13].

The DIGIHOME middleware platform is proposed in [14] in order to support the integration of pervasive environment devices. The integration of heterogeneous devices is based on REST principles; however, the dynamic discovery of resources in this middleware is dependent on UPnP and Service Location Protocol (SLP). In contrast, we use the RESTful approach for service discovery and eventing.

There have been some efforts for standardizing AAL infrastructures such as the UniversAAL open platform [15] and Continua Alliance [16], which works on enabling complete health care and fitness systems, and focuses on achieving interoperability among health care companies and devices. Nevertheless, a recent survey of RESTful web services for service provisioning in next-generation networks [5] identified the potential of RESTful web services for interconnecting heterogeneous devices and services. However, the authors also iterated that no appropriate dynamic service publication and discovery platform for REST has been defined yet. In this paper, we address this problem and propose a solution for it.

## 3. RESTful Approach for Discovery and Eventing

### 3.1. REST Basics and Potentials

REST is an architectural style, outlined and proposed by Fielding, which defines four design principles for distributed hypermedia systems: (P1) unique identification of resources; (P2) uniform interface to manipulate resources through representations; (P3) self-descriptive messages; and (P4) hypermedia as the engine of application state [17]. The modern web is one instance of a REST-style architecture and can be used to briefly explain the above four principles. They essentially define a resource oriented abstraction where, everything in the web is a resource that could be uniquely addressed, and could be accessed using a uniform interface.

For example, “http://www.mdpi.com/journal/sensors/special_issues/aal” is an URI that identifies and addresses a resource on the web (P1) and its representation is an HTML document, which can be retrieved by means of an HTTP GET request (P2). This request message includes all the required information to get the resource representation in the URI and in the HTTP headers (e.g., accepted content-type) and hence, the server does not rely on previous requests (statelessness) to send the response, which includes HTTP status code and additional HTTP headers along with the resource representation (P3). The HTML document contains hyperlinks to other resources, which are used to guide the client through the application without maintaining any application state in the server (P4).

HTTP servers can host a number of resources uniquely identified by URIs. However, assuming that resources are related to files in the server file system, is a common misunderstanding (simplified view of a web server). Resources can represent other entities than that of files and can be generated dynamically. When a request for a given resource arrives to the HTTP server that is hosting the resources, it decides how the resource identifier is mapped down to the actual computing entities that implement the resource [18].

In addition to only GET and POST methods, HTTP/1.1 defines other methods such as DELETE, PUT, OPTIONS, HEAD, TRACE and CONNECT, each of which has an associated semantics with respect to the target resource and conform the uniform interface in a RESTful architecture. Here, DELETE may be used to remove the resource identified the URI; PUT may be used to create or update the resource representation; OPTIONS can be used to obtain the set of methods supported by the resource; HEAD can be used to retrieve a metadata-only representation of the resource; while TRACE and CONNECT are very rarely used and have no special interest in RESTful architectures.

Furthermore, HTTP/1.1 also specifies a set of status codes that provides a very rich set of semantics for the message exchange. For example, a “200 OK” status code indicates a successful HTTP request, while a “201 Created” status code indicates that the creation request has been fulfilled and resulted in a new resource being created. Both of these status codes indicate successful actions but have separate semantics. Similarly, there are other status codes that can be leveraged for relevant purposes. Beside the status codes, HTTP/1.1 defines the HTTP headers that specify internals and features of the data exchange, ranging from the accepted or provided content-type to conditional request expressions based on resource modification date. The combined and intensive use of HTTP methods, status codes and headers provide a powerful and rich uniform interface for designing RESTful applications and services [18].

Similar to the web resource representation, REST principles can also be applied to define and represent networked devices and services and the communications among them. Inherently, a group of sensors and actuators is a set of distributed resources. Therefore, considering and representing the devices as REST resources is not a surprising approach. Thus, a sensor such as a weight scale could be represented as a resource, identified by a URI like “http://myHome/weight_scale“. The measured weight and the current user can also be represented by two other resources identified by “http://myHome/weight_scale/weight” and “http://myHome/weight_scale/user”, respectively. So if we consider the weight resource, sending a GET request to the weight resource should retrieve the last weight measurement of the weight scale. While, if the objective is to set the user that is using the scale, a PUT message containing the user identification can be sent to the “user” resource, which will update the user representation.

### 3.2. Discovery

#### 3.2.1. Background on Discovery

**Figure 1.**
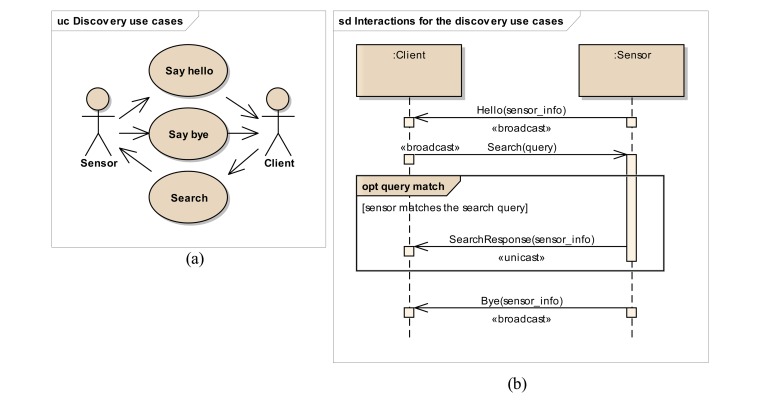
Discovery use cases (**a**) and behavior (**b**).

Discovery protocols specify the rules and behaviors required to advertise and locate services (e.g., sensors or other devices) in a networked environment. There are typically three use cases in discovery mechanism as shown in Figure 1a. Services can advertise themselves by broadcasting messages with their description and location, so that potential clients can be aware of them. Services can send these advertisements either for announcing their presence (Say hello) or for communicating their unavailability (Say bye). Additionally, clients (e.g., an application or another service) can search for target services, by means of broadcasting a query message (Search) to announce their interests and waiting for replies from matching services. Technologies such as WS-Discovery (DPWS) [19] and SSDP (UPnP) [20] represent existing discovery specifications that can be applied to networked environments. This paper introduces a REST based discovery specification.

Figure 1b shows the interactions between clients and sensors in the mentioned discovery use cases. Sensors broadcast a Hello message containing their own information and location (Say hello use case) to the listening clients. On the other hand, clients can also broadcast messages for querying specific sensor types (Search use case). The matching sensors will reply to this query request by sending a unicast message to the client, describing themselves and providing their network location. When a sensor is no longer available in the network, it broadcasts a Bye message (Say bye use case) to the network so that all the clients can react accordingly.

#### 3.2.2. Discovery Model Specification

The fundamental of the proposed discovery model is based on defining a list that should contain all the available services in the network. In order to conceptualize this, we define a virtual resource that represents the list. The URI of this virtual resource is “http://224.100.0.1:28888/Resources“ which belongs to a multicast group. Any sensor or client application can join in this group and, thus, be aware of changes in the virtual list of resources. This abstraction that represents a list of available resources is the basis of the discovery mechanism. When a new sensor comes into the network it is included in the virtual list, and when an existing sensor leaves the network, it is removed from the list.

It must be remarked that this list does not really exist and is just a virtual artifact defined for supporting the discovery model. The remainder of this section describes the specific HTTP messages that must be sent to this virtual resource in order to implement the discovery use cases identified in Figure 1a in a RESTful way. All the messages to the multicast group are based on HTTP over UDP.

**Figure 2.**
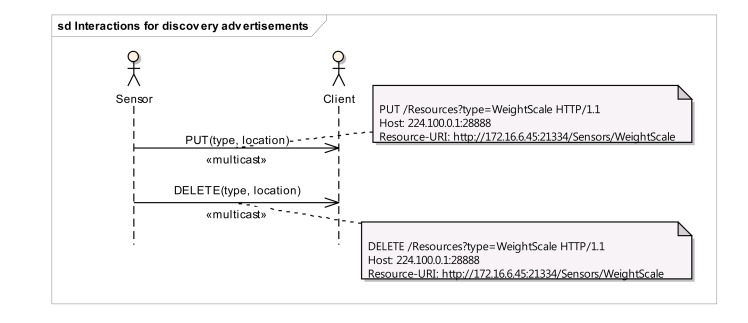
Sequence diagram for sensor advertisement.

The interactions for sensor advertisement are depicted in Figure 2. Sensors advertise their presence (Say hello) by sending a multicast PUT message to the virtual “http://224.100.0.1:28888/Resources” resource, describing their own type and location. The figure shows an annotation with a sample message that illustrates how a weight scale device (type = “WeightScale”, location = “http://172.16.6.45:21334/Sensors/WeightScale” ) would advertise itself to potential listeners in the multicast group. For the sake of clarity, the advertised sensor type (WeightScale) is described using a simple textual description, although more complex types could also be applied. In a similar way, when the sensor leaves the network (Say bye use case), it sends a multicast DELETE message to the virtual resource. This message will reach to all the group members indicating the unavailability of the sensor in the network, so that the group members can react to this change.

**Figure 3.**
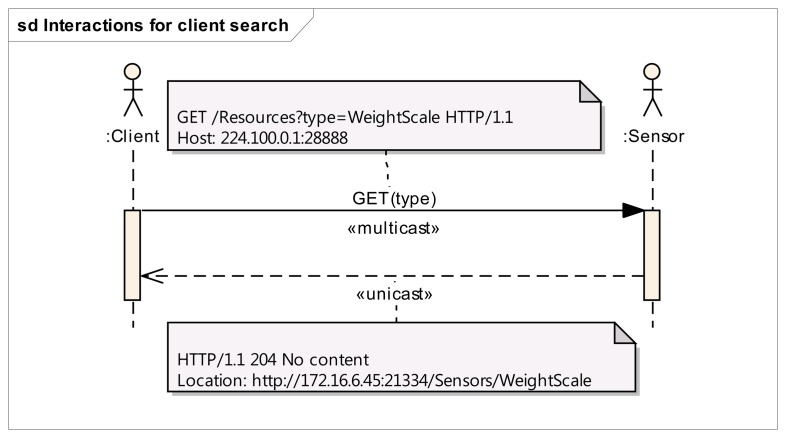
Sequence diagram for client search.

The interactions required for the “Search” use case are described in Figure 3. A client can search for a specific sensor type by sending a multicast GET message to the virtual resource, which indicates the type of the sensor it is interested in. All the sensors that match the request shall reply to it by sending a unicast message to the client containing the sensor location. This unicast response is sent using HTTP over UDP. If the client does not specify any type in the GET request, all the sensors in the network shall reply to this query. The annotations in the sequence diagram show the sample messages interchanged between a client (who is searching for a “WeightScale”) and the matching sensor (which sends its location URI to the client).

Table 1 contains the specification of the messages we just described. The third column defines message templates for each use case that must be adjusted by replacing {resource type} and {URI} with representative value.

### 3.3. Eventing

#### 3.3.1. Background on Event Management

Event notification mechanisms are typically based in the observer pattern, whose purpose is the definition of a one-to-many dependency between objects so that when the base object changes state, all its dependents are automatically notified. The pattern establishes a simple model to define the relationships among the participants to achieve the desired publish-subscribe capabilities. In a general eventing model we can distinguish the following elements: event sources, event listeners, subscriptions and notifications. Event sources are the event generators that post the event information. Event listeners have the ability to express interest in an event. Subscriptions represent client interest on events and are accepted by event sources. Finally, a notification is the message that the event source sends to the event listener containing the event information. There are several proposals that can be applied to event notification. WS-Eventing (DPWS) [19] and GENA (UPnP) [20] are two eventing specifications that can be applied to an AAL environment.

**Figure 4.**
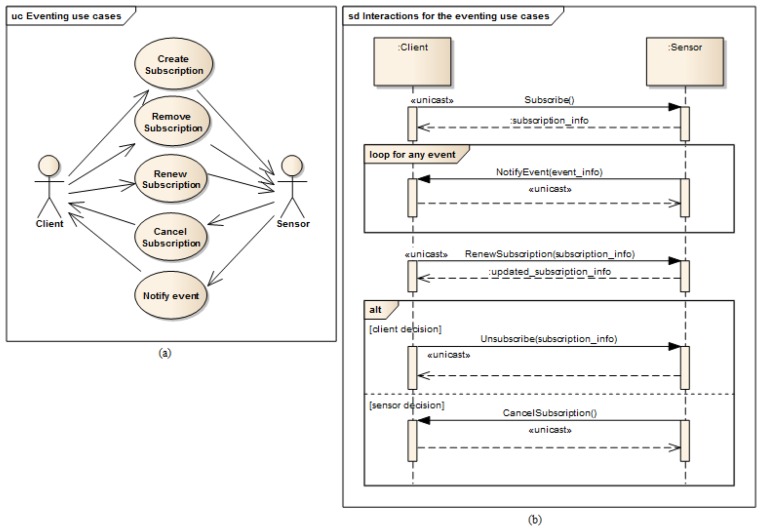
Event management use cases (**a**) and behavior (**b**).

Figure 4a represents the use cases of event management mechanism, where the Client actor represents “event listeners” and the Sensor actor represents “event sources”. Figure 4b shows the high level interactions between client and sensor for the eventing use cases. Initially (Create Subscription use case), the client subscribes to listen to some event of the sensor and the sensor replies with the newly created subscription information (*i.e.*, unique identifier for the subscription and expiration time) that should be used in further interactions regarding this subscription. Later, any change in sensor data will be sent (Notify Event use case) to the client. The subscription can be renewed (Renew subscription use case) in order to listen to events for extended period. Finally can be destroyed when the client is no longer interested in listening to events (Remove subscription use case) or when the sensor is not able to notify the changes of its data (Cancel subscription use case). All the described interaction messages are exchanged using “unicast” mechanism between the communicating parties.

#### 3.3.2. Proposed RESTful Eventing Model Specification

**Figure 5.**
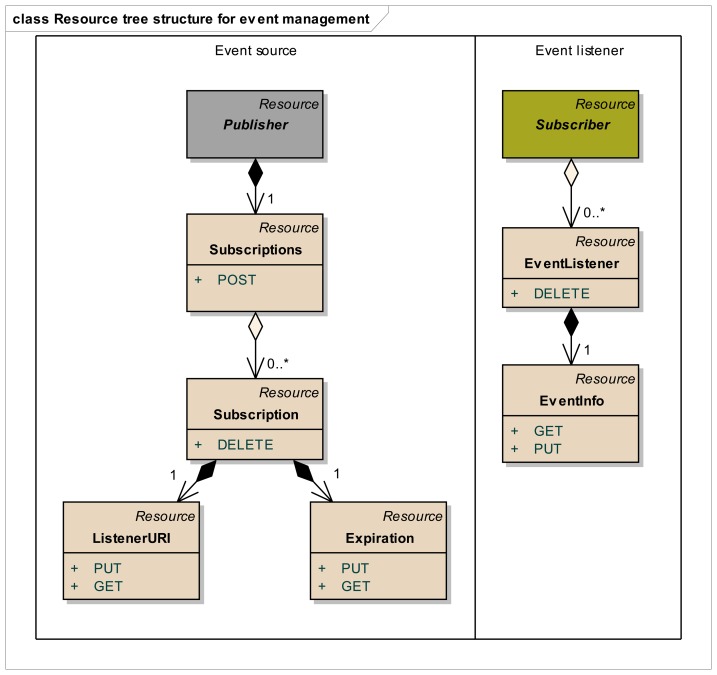
Resource model for event management.

**Figure 6.**
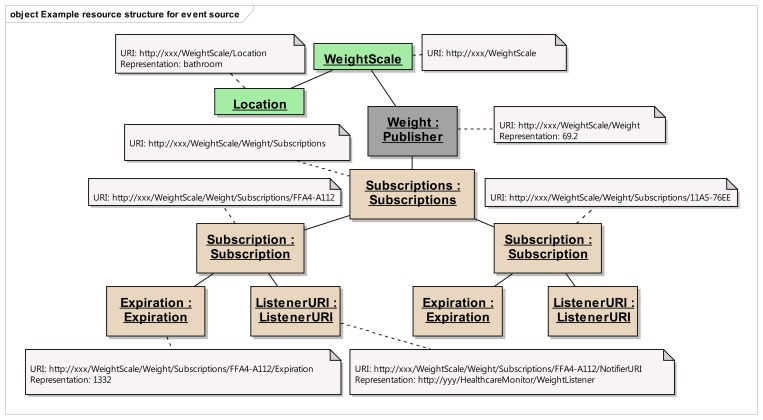
Resource tree for a weight scale with annotations for URI and representations.

**Figure 7.**
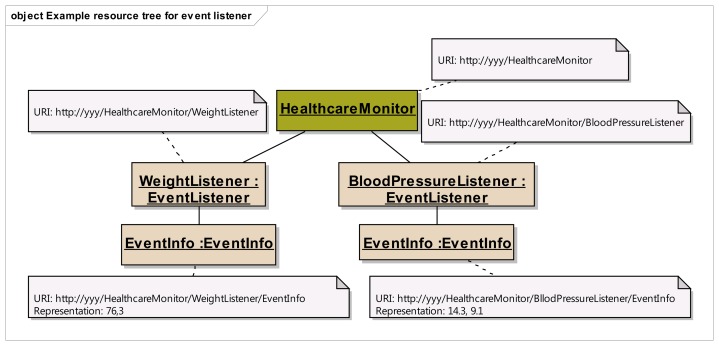
Resource tree for a healthcare monitor with annotations for URI and representations.

**Figure 8.**
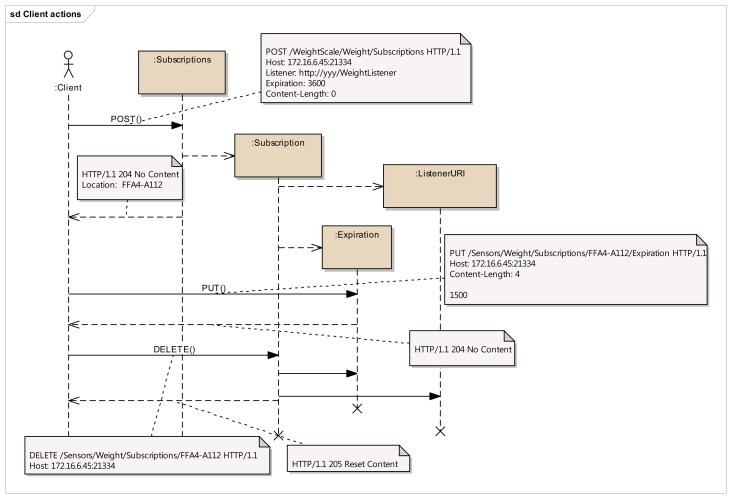
Interactions between resources of event source.

**Figure 9.**
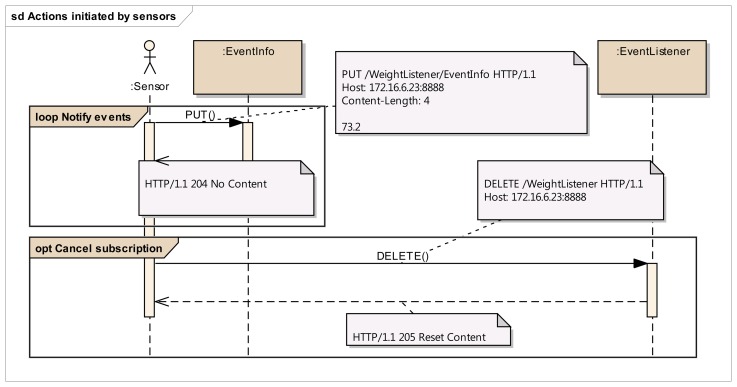
Interactions with resources of event listener.

The proposed RESTful eventing model specification is described in several steps. They are as follows:

Step 1: Identification of resource structure to represent event sources and event listeners
  Based on the event management use cases given in Figure 3, the required resource structure to represent event listeners and event sources is identified. The core of this structure consists of two resource trees, as depicted in Figure 5, one representing the event source, and the other representing the event listener. The resource tree structure of the event source represents the public view for the event listeners, whereas the resource tree structure of the event listener represents the public view for the event sources.
  Event source tree is composed of an abstract “Publisher” resource which contains a “Subscriptions” resource, representing a container for the list of potential subscriptions, each of them being represented by the “Subscription” resource. A “Subscription” resource consists of a “ListenerURI” resource and an “Expiration” resource.
  On the other hand, the event listener tree is composed of an abstract “Subscriber” resource which can integrate many “EventListener” resources, each of them containing an “EventInfo” resource. A single “EventListener” resource can listen to a single event source. An “EventInfo” resource represents the event data notified by the event source.
  The specification and the semantics of the methods (GET, POST, PUT and DELETE) used for the resources in Figure 5 are detailed in Table 2 (for event source) and Table 3 (for event listener).
  The methods within the resource map the interactions required for the identified use cases in the event management. In these tables, the first column lists all the required resources, second column refers to representation, third column lists all the supported methods and the fourth column shows the specific HTTP headers to parameterize the requests and the responses. For the responses in fourth column, the HTTP status code is shown.
Step 2: Resource tree instantiation for sample event source and event listener
  Figure 6 shows the resource tree for a sample healthcare device (a weight scale) that has some data whose changes could be of interest to potential listeners. The main resource (WeightScale) represents the device that has two attributes (represented by resources as well), such as the “Location” and “Weight”. Some of these attributes can have events, adopting the Publisher role. It must be noted that this role is not taken by the “WeightScale” resource, but by the “Weight” resource, because the events shall be raised on changes of the measured weight, which is the representation of this resource (e.g., 69.2 kg). Thus, the corresponding “Subscriptions” resource shall belong to the “Weight” resource, not to the “WeightScale” resource. It should be clear from this figure, that the “Weight” resource is an instantiation of the abstract “Publisher” resource in Figure 5, and that two different listeners represented by the corresponding “Subscription” resources are subscribed to the “Weight” of the scale.
  The URIs and representations of the relevant resources are also annotated in Figure 6. For the sake of simplicity, in this example, we are not considering complex representations of the resources (e.g., XML, JSON, etc.) but just a simple text/plain content type.
  In a similar way, Figure 7 shows an example of the resource tree that a healthcare monitor application (event listener) would expose in order to receive notifications from the corresponding sensors (in this case, a weight scale and a blood pressure monitor). The “HealthcareMonitor” resource is the root of the subscriber application, containing multiple event listener resources. Thus, the “WeightListener” and “BloodPressureListener” resources are instances of the EventListener class shown in Figure 5, with their corresponding “EventInfo” child resource, which represents each specific event data. Again, simple text/plain types have been used for event data.
Step 3: Interactions with resources of event source
  Figure 8 depicts the interactions initiated by a client that occur in the subscription management process. Any client who is interested in getting a notification when a new weight measurement is taken, shall POST a message to the “Subscriptions” resource of the “Weight” resource. This message shall include both the URI where the sensor should send the notifications for the client to listen (e.g., http://yyy/WeightListener) and the desired expiration time (3600 s), using specific HTTP headers. On the reception of this request, the sensor shall create a new unique Subscription resource for this specific client, storing the listener URI and the expiration time creating the corresponding resources. In the reply for the subscription request, the sensor shall include the name of the new subscription (e.g., FFA4-A112) using the Location HTTP header, thus allowing the client to identify the URI of the dynamically created resource. This URI will be used by the client and the sensor in further interactions related to the subscription management (renewing, removing or cancelling the subscription).
  Whenever the clients decides to renew the subscription, it shall update the representation of the expiration time, by means of sending a PUT request to its subscription's Expiration resource, which will be replied by the sensor acknowledging the update.
  If the client decides to remove the subscription because it is no longer interested in receiving updates of the weight measurement, it shall simply send a DELETE request to the Subscription resource, indicating to the sensor that this subscription is no longer required. The sensor shall reply accordingly.
Step 4: Interactions with resources of event listener
  In Figure 9, the interactions that occur between a sensor and the resources of an event listener resources. Whenever a sensor data changes, the sensor will send a PUT message to the specific “EventInfo” resource of all the event listeners, containing the specific representation of the event data. A simplistic representation of the event data is shown in the annotation of this diagram (a basic text/plain type) for the sake of clarity, though more complex representations could be used. Finally, in the case the sensor can no longer support the subscription, it will notify this to the subscriber) sending a DELETE message to the specific “EventListener” resource of the client.

## 4. Evaluation

The goal of this section is to evaluate and demonstrate the proposed approach. To do so, we first compare the RESTful solution with two other dominant technologies currently being used for discovery and eventing. We then show the suitability of the proposed solution by implementing it in the context of an AAL healthcare scenario. In the following we describe these two objectives.

### 4.1. Comparing REST with DPWS and UPnP

We compare the proposed RESTful approach with DPWS and UPnP in terms of the size of the messages that are required for dynamic discovery and eventing. The comparison is done based on the specification of these technologies. We measure the size of each specific message that is required for the different uses cases identified in discovery and eventing. The efficiency in terms of processing time needed for message composing, parsing and networking is highly related to the message size.

**Figure 10.**
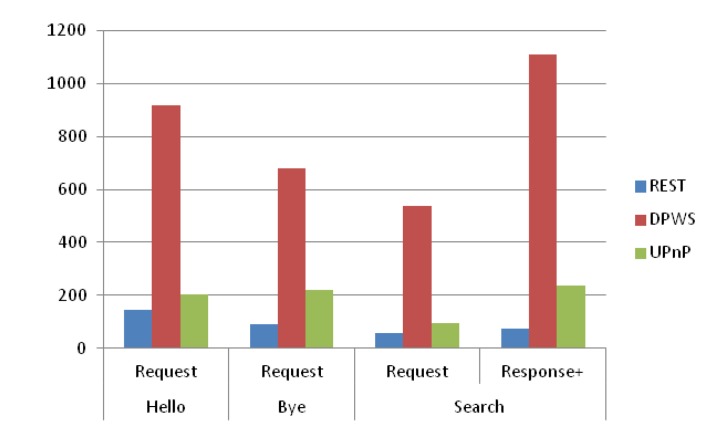
Comparison of message size (bytes) for discovery use cases.

In the case of discovery, Figure 10 compares the message sizes for Hello, Bye and Search use cases as they are defined in the three targeted solutions (REST, DPWS, UPnP). The results shown in this figure are based on single message size for each type, without considering the multiple repetitions that are mandatory for the multicast messages used in Hello, Bye and Search request messages. Thus, if we consider the required repetitions, the accumulated message size of the RESTful approach will be much smaller than the other studied approaches, which demonstrates its lightweightness. In the case of DPWS, the overhead is due to the use of SOAP and XML format in the message specification. The main difference between UPnP and REST results comes from the additional description needed to express the semantics of the Hello and Bye messages. UPnP does not benefit from the semantics of HTTP methods because the same NOTIFY method is used for both Hello and Bye messages requiring additional data definition (NTS:ssdp:byebye and NTS:ssdp:alive headers) instead of simply using PUT (hello) and DELETE (bye) methods.

**Figure 11.**
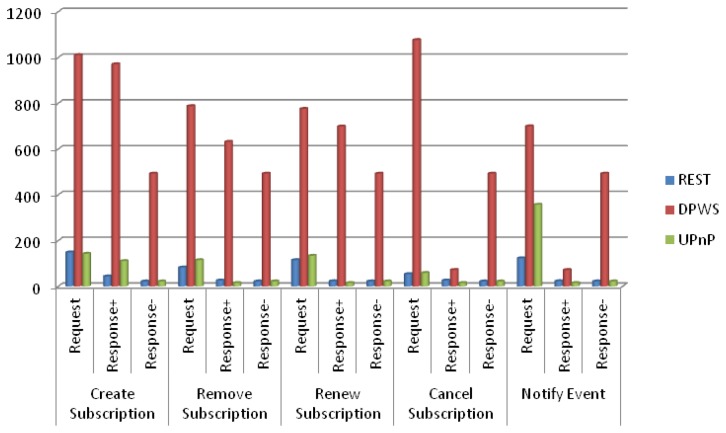
Comparison of message size (bytes) for eventing use cases.

The same analysis has been done for the messages involved in the eventing use cases, as shown in Figure 11, and it's visible that the messages in the RESTful approach are smaller in size than the messages used in other solutions. All the messages are unicast, without no need of repetitions, and we have considered REQUEST, RESPONSE+ for correct responses and RESPONSE- for faulty responses (generated when sending incorrect requests). SOAP and XML are still an issue for DPWS, while REST and UPnP have similar message size except in the case of Notify Event. In this case, UPnP specifies the using of a very constrained XML schema for event data representation (only the single value) and further, can not use more efficient formats such as plain text or JSON.

Overall, the RESTful approach is lightweight and more efficient that the others because due to the smaller message size, as we demonstrated, the message composing, parsing and networking takes less time.

### 4.2. Implementation Infrastructure of Prototype

**Figure 12.**
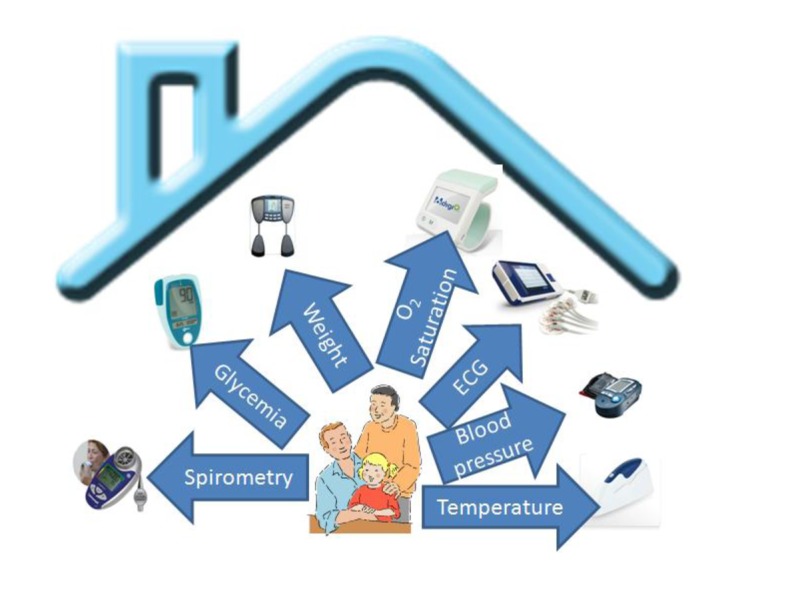
Healthcare sensors used in the AAL environment.

In order to investigate the suitability of the proposed approach in AAL, this section describes a prototype pervasive application for healthcare support in a home environment that has been implemented using the RESTful discovery and eventing specifications, proposed in this paper. Nowadays several health monitoring sensors are available in the market with heterogeneous networking capabilities. We took a set of representative sensors and embedded them in an AAL environment in order to capture different physiological parameters like blood pressure, O_2_ saturation, weight, temperature, glycemia, cholesterol, electrocardiogram (ECG) and spirometry, as shown in Figure 12.

This prototype realizes a monitoring system that seamlessly captures and stores data from a set of heterogeneous health sensors as mentioned before, displaying elaborated health information to users by means of a front-end application, running in a SmartTV and a tablet.

**Figure 13.**
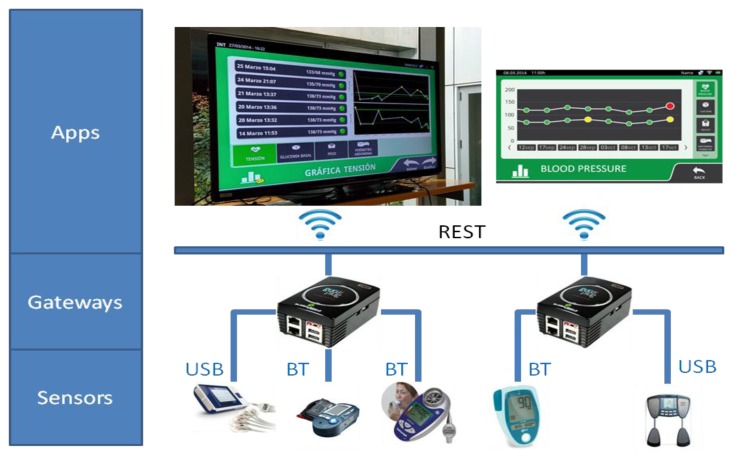
Demonstrating application deployment.

The prototype demonstrates a distributed architectural deployment of the healthcare application as shown in Figure 13. The sensors layer is composed of a number of devices with heterogeneous communication capabilities and device-dependent protocols such as USB and Bluetooth serial communication profiles.

It is possible that a sensor device implements the proposed mechanism in order to support dynamic discovery and eventing policies in a RESTful way, however none of the sensors used in this prototype had those capabilities. Without this support, the devices need to be integrated using a different mechanism, such as the one we adopted with the help of several plug computers running Debian/Linux operating system. We then built a Java prototype implementation of the RESTful service discovery and eventing model which acts as a gateway from the device proprietary protocols to a Wi-Fi network, exposing all the health devices as REST resources that can be discovered and subscribed by any potential application.

The Application layer in the figure is developed as a Java based user interface, that tracks and monitors data coming from the available sensors, running in Android home devices (SmartTV and tablet). The application is built on top of a homogeneous middleware, a prototype of the proposed approach, which offers discovery and eventing mechanisms, thereby isolating the application from the variability of underlying device protocols. The specific set of hardware components used for this prototype implementation is listed in Table 4.

## 5. Conclusions

This paper proposed a RESTful discovery and eventing specification, which is a lightweight and efficient approach compared to existing service discovery and event management solutions. To the best of our knowledge this is the first RESTful approach in this direction. This work is described by specifying the RESTful resource tree structure, resource tree instantiation for sample event source and event listener, and interactions between resources of event source and event listener for both discovery and eventing mechanisms. We evaluated the proposed approach with existing ones as per specification and found that the RESTful approach needs minimal message size for service discovery and eventing, which is very promising. We implemented the proposed technique in the context of an assisted living healthcare scenario for connecting heterogeneous sensor and actuator devices in order to obtain real-time health data from the inhabitants for better health monitoring. The proposed approach described in this paper would be a suitable solution for many pervasive sensor-rich environments for dynamic service provisioning. Therefore, in the future we plan to demonstrate the applicability of our approach in various pervasive applications domain, and to study the requirements for safety and security that come together with the use of health related data.

## Figures and Tables

**Table 1.** Message templates for discovery use cases.

**Use Case**	**Method**	**Message Template**	
Say hello	PUT	PUT/Resources?type= {resource type} HTTP/1.1	
		Host: 224.100.0.1:28888	
		Resource-URI: {URI}	

Say bye	DELETE	DELETE/Resources?type= {resource type} HTTP/1.1	
		Host: 224.100.0.1:28888	
		Resource-URI: {URI}	

Search	GET	REQUEST	GET/Resources[?type= {resource type} ] HTTP/1.1
			Host: 224.100.0.1:28888

		RESPONSE	HTTP/1.1 204 No content
			Location: {URI}

**Table 2.** Publisher's resource specification detail.

**Resource**	**Representation**	**Method**	**Required Headers**	
Subscriptions	None	POST Creates a new subscription	REQUEST	Host: {Host domain info}
				Listener: {URI}
				Expiration: {Seconds}
				Content-Length: 0

			RESPONSE 204 No content	Location: {URN}

Subscription	URN	DELETE Removes a subscription	REQUEST	Host: {Host domain info}

			RESP ONSE 205 Reset content	

ListenerURI	URI	PUT Updates the listener URI	REQUEST	Host: {Host domain info}
				Content-Type: text/plain
				Content-Length: {length}

			RESPONSE 204 No content	

		GET Obtains the listener URI	REQUEST	Host: {Host domain info}

			RESPONSE 200 Ok	Content-Type: text/plain
				Content-Length: {length}

Expiration	Number of seconds	PUT Updates the expiration time	REQUEST	Host: {Host domain info}
				Content-Type: text/plain
				Content-Length: {length}

			RESPONSE 204 No content	

		GET Obtains the remaining expiration time	REQUEST	Host: {Host domain info}

			RESPONSE 200 Ok	Content-Type: text/plain
				Content-Length: {length}

**Table 3.** Subscriber's resource specification detail.

**Resource**	**Representation**	**Method**	**Required Headers**	
EventListener	None	DELETE Cancels a subscription	REQUEST	Host: {Host domain info}

			RESPONSE 205 Reset content	Location: {URN}

EventInfo	Event data	PUT Notifies an event	REQUEST	Host: {Host domain info}
				Content-Type: {content-type}
				Content-Length: {length}

			RESPONSE 204 No content	

		GET Obtains the last event	REQUEST	Host: {Host domain info}

			RESPONSE 200 Ok	Host: {Host domain info}
				Content-Type: {content-type}
				Content-Length: {length}

**Table 4.** Integrated devices for healthcare application.

**Devices**	**Model**
Blood pressure	Taidoc TD-3132
O_2_ Saturation	DigiO_2_ HemOxi POM-101
Weight	Tanita BC-590BT
Glycemia	Taidoc TD-4255
ECG	Taidoc TD-2202
Spirometry	Vitalograph copd-6
Temperature	Taidoc TD-1261A
Plug computer	Globalscale GuruPlug Server
SmartTV	Sony NSZ-GS7
Tablet PC	Archos 80 G9
